# Attitudes toward pre-symptomatic screening for Alzheimer’s dementia in five European countries: a comparison of family members of people with Alzheimer’s dementia *versus* non-family members

**DOI:** 10.3389/fgene.2023.1305107

**Published:** 2023-12-15

**Authors:** Ioanna A. Angelidou, Marina Makri, Konrad Beyreuther, Mercè Boada Rovira, Akyllina Despoti, Sebastiaan Engelborghs, Andrea Miguel, Isabel Rodríguez, Hannah Stocker, Joke Temmerman, Magda Tsolaki, Görsev Yener, Deniz Yerlikaya, Birgit Teichmann

**Affiliations:** ^1^ Network Aging Research (NAR), Heidelberg University, Heidelberg, Germany; ^2^ 1st Department of Neurology, School of Medicine, Faculty of Health Sciences, Aristotle University of Thessaloniki, Thessaloniki, Greece; ^3^ Greek Association of Alzheimer Disease and Related Disorders, Thessaloniki, Greece; ^4^ Laboratory of Neurodegenerative Disease, Center for Interdisciplinary Research and Innovation (CIRI—AUTh), Balkan Center, Aristotle University of Thessaloniki, Thessaloniki, Greece; ^5^ Research Center and Memory Clinic, Ace Alzheimer Center Barcelona–Universitat Internacional de Catalunya, Barcelona, Spain; ^6^ Clinical Ergospirometry, Exercise and Rehabilitation Lab, School of Medicine, National and Kapodistrian University of Athens, Zografou, Greece; ^7^ Department of Neurology and NEUR Research Group, Center for Neurosciences, Universitair Ziekenhuis Brussel and Vrije Universiteit Brussel, Brussel, Belgium; ^8^ Faculty of Medicine, Izmir University of Economics, Izmir, Turkiye

**Keywords:** Alzheimer’s disease, screening attitude, perceived harms, perceived benefits, cultural differences

## Abstract

**Introduction:** Pre-symptomatic screening is getting more attention in healthcare as it detects the risk for developing neurodegenerative diseases like Alzheimer’s disease (AD), which is very useful for treatment or prevention. AD screening could play an important role in individuals with at least one affected first-degree relative, but also without family history. As the demand for screening is rising worldwide, it is important to consider possible cross-cultural differences in attitudes toward pre-symptomatic screening in order to tailor healthcare services to the needs of each country.

**Objective:** This study aims to investigate the attitudes of family members and non-family members of people with dementia toward pre-symptomatic screening and explore possible differences in attitudes across five European countries (Belgium, Germany, Greece, Spain, Turkey) using translated versions of the “Perceptions regarding pRE-symptomatic Alzheimer’s Disease Screening” questionnaire (PRE-ADS).

**Methods:** The multicultural sample (N = 650) was recruited from samples that were previously used in validation studies of the translated PRE-ADS versions. The subscale “Acceptability of Screening”, consisting of five PRE-ADS items to specifically explore willingness to undergo screening, was created. Ιnternal consistency was measured, and structural validity was determined using Confirmatory Factor Analysis (CFA). Group comparisons were performed to investigate differences in attitudes toward pre-symptomatic AD screening regarding family history and country of origin using the PRE-ADS and the “Acceptability of Screening” mean scores.

**Results:** Construct validity was acceptable for the PRE-ADS. Both the PRE-ADS (*α* = 0.76) and its subscale “Acceptability of Screening” (*α* = 0.90) had good internal consistency. Overall, 56.9% of the total sample expressed a positive intention toward pre-symptomatic AD screening. T-tests showed significantly higher mean scores of participants with an affected family member. An international comparison revealed differences in the “Acceptability of Screening” mean score across the five European countries. No cross-cultural differences were found for the PRE-ADS mean score after adjusting for confounding variables.

**Conclusion:** The PRE-ADS and its subscale are reliable tools for assessing pre-symptomatic AD screening attitudes. Variations in the acceptability of screening seem to be linked to family history and cultural influences. Further research with larger samples is needed to explore underlying relationships.

## Introduction

Alzheimer’s disease (AD) and related dementias account for 60%–70% of all dementia cases and are the most common neurodegenerative disorders. AD is characterized by progressive cognitive decline leading to memory loss, disorientation, behavioral abnormalities, and, ultimately, the inability to live independently (Hansson, 2021; Hu et al., 2022). The continuous aging of the global population is expected to lead to an increase in the number of people with dementia from 50 million today (GBD, 2016 Dementia Collaborators, 2019) to 135 million by 2050 (Prince et al., 2015), with almost 19 million cases in Europe (Alzheimer Europe, 2013; Prince et al., 2015). The prevalence of dementia is increasing despite the decline in age-specific incidence in European populations, which is most likely due to preventive measures and improved pharmacological treatment options for cardiovascular and cerebrovascular risk factors (Frisoni et al., 2023). Dementia is an enormous public health issue, the cost of which could become a major social problem (Hu et al., 2022). Even if disease-modifying therapies become a reality (Li et al., 2022), delaying the onset or progression of the disease through appropriate preventive measures will remain of utmost importance. Brookmeyer et al. (2007) calculated that delaying the onset of dementia by 1 year would result in almost 9.2 million fewer cases in 2050, drastically reducing the number of people needing care. Therefore, early detection of neuropathologic changes that may occur 10–15 years before clinical symptoms are visible is becoming increasingly important (Mahaman et al., 2022).

While the pathophysiology of AD is complex and still not fully understood, it is characterized by *β*-amyloidosis and neurofibrillary tangles (NFTs) (Varesi et al., 2022), as well as neurodegeneration, synapse loss, and associated neuroinflammation (Mahaman et al., 2022). AD biomarkers, which act as a proxy for amyloid plaques, neurofibrillary tangles and neurodegeneration, can be assessed using biofluid (cerebrospinal fluid (CSF), plasma) and imaging markers (e.g., brain MRI scan or amyloid-, tau- and 18F-fluorodeoxyglucose (FDG)-PET scan of the brain) (Mahaman et al., 2022). Recent trials have shown that new drugs can reduce brain amyloid levels, which were associated with a modest reduction in cognitive decline compared with a placebo group, but more research is needed as this therapy is associated with adverse events (van Dyck et al., 2023). It is, therefore, important to detect the pathology of AD at an early stage, when disease-modifying drugs may be more promising. While AD biomarkers screen for ongoing pathology, risk calculation can also be based on genetic testing for genes associated with familial AD (FAD) or late-onset AD (LOAD). Rare autosomal dominant mutations in amyloid precursor protein (APP) and presenilin 1 and 2 (PSEN1 and 2) with virtually 100% penetrance account for 80% of FAD cases, with disease onset usually before the age of 60. Mutations in PSEN1 on chromosome 14q24.3 account for at least 50% of all cases, whereas mutations in APP on chromosome 21q21 account for another 10%–15%, and mutations in PSEN2 on chromosome 2q31-q42 are rare, except in families of Volga German ancestry (Sherrington et al., 1996; Jayadev et al., 2010). A much higher proportion of AD is due to LOAD, with polygenic risk from multiple susceptibility genes. Although genome-wide association studies (GWAS) have identified more than 40 risk alleles for AD, *APOE*, located on chromosome 19q13.2 (Karch et al., 2014), confers a significantly higher risk than any other gene locus and accounts for 20%–29% of AD. *APOE* has three different allelic forms, ε2, ε3 and ε4, which give rise to the possible genotypes: APOE ε2/ε2, ε2/ε3, ε2/ε4, ε3/ε3, ε3/ε4 and ε4/ε4. The ε3/ε4 genotype increases the lifetime risk of developing AD by a factor of 2–3, while those with ε4/ε4 have an increased risk up to a factor of 15 (Goldman, 2012).

There are two ways of identifying people at risk for dementia: a population-based approach, which would need to be inexpensive and non-invasive, or an approach that identifies the group at risk. As the latter involves a smaller group of people, it may be more costly, invasive, and/or inconvenient (Gordis, 2014). Population-based screening with blood-based biomarkers could be a solution to identify those who need more costly tests and to implement personalized treatment or prevention interventions (Ribaldi et al., 2019). However, current guidelines, such as those from the US Preventive TaskForce (USPSTF), do not recommend population-based screening at this time because more research is needed to assess the balance of benefits and harms of screening for cognitive impairment in older adults (Owens et al., 2020). This is in accordance with Sackett’s guideline, which asserts that “screening for a disease is appropriate when available screening tests are acceptable to patients, when the treatment early is more beneficial than treatment later in the illness, and when time and resources are sufficient to allow screening, diagnosis, and treatment of the disorder” (Sackett et al., 2006).

According to a systematic review of Martin and others (2015), studies on population screening have shown that attitudes and preferences are multifactorial and that caution is warranted until the benefits and risks have been thoroughly investigated. In addition, no study has been conducted on a representative sample, so the evidence is weak (Martin et al., 2015).

While some studies on dementia screening have examined acceptability, perceived harms, or perceived benefits (Justiss et al., 2009; Boustani et al., 2011; Holsinger et al., 2011; Fowler et al., 2012; Braun et al., 2014; Fowler et al., 2015; Martin et al., 2015), there are few studies on attitudes toward pre-symptomatic screening. The “Perceptions regarding pRE-symptomatic Alzheimer’s Disease Screening” (PRE-ADS) (Makri et al., 2023) is a further development of the Perceptions regarding “Investigational Screening for Memory in Primary Care” (PRISM-PC) questionnaire and measures attitudes, motivations, and barriers to pre-symptomatic screening for dementia. Thereby, acceptability usually refers to the degree to which individuals or communities find a particular health intervention suitable, agreeable, and appropriate (Becker, 1974). Attitude is composed of a cognitive, a behavioral, and an affective component (Rosenberg, 1960) and encompass individuals’ thoughts, feelings, and evaluations regarding pre-symptomatic screening. Motivations refer to differential emotional arousal and the driving forces or reasons that prompt individuals to undergo pre-symptomatic screening, caused by some given class of stimuli (Becker, 1974), while barriers can be referred to as obstacles, challenges, or perceived “costs” (Becker, 1974) that hinder the acceptance or implementation of pre-symptomatic screening.

The PRE-ADS has been translated and validated in the following languages: Greek (Cronbach’s *α* = 0.82) (Makri et al., 2023), German (Cronbach’s *α* = 0.78) (Angelidou et al., 2023), Dutch (Cronbach’s *α* = 0.75), Spanish (Cronbach’s *α* = 0.83), and Turkish (Cronbach’s *α* = 0.69) (Table 1). Studies of pre-symptomatic genetic testing for dementia susceptibility genes have been conducted primarily in first-degree family members. For example, Kopits et al. (2011) examined willingness to pay for genetic testing, while the Risk Evaluation & Education for Alzheimer’s Disease (REVEAL) study primarily examined the psychological effects of genetic testing for *APOE* genotype status in successive, multi-site trials to provide insight into the potential benefits or harms of risk disclosure (Chao et al., 2008; Ashida et al., 2010; Besser et al., 2015; Roberts, 2019).

**TABLE 1:** Results of previous validation studies of the PRE-ADS and translated versions.

Country	Version	α**	Factor structure	Items	*α*
Greece^∗^	PRE-ADS (Makri et al., 2023)	0.82	1 “Perceived Harms of Testing”	*n* = 10 (9, 10, 11, 12, 15, 16, 18, 19, 20, 21)	0.87
			2 “Acceptance of Testing”	*n* = 5 (1, 2, 3, 4, 5)	0.86
			3 “Perceived Benefits of Testing”	*n* = 6 (13, 17, 22, 23, 24, 25)	0.76
			4 “Need of Knowledge”	*n* = 4 (6, 7, 8, 14)	0.70
Belgium	PRE-ADS (Dutch version)	0.75	1 “Perceived Harms of Testing	*n* = 10 (9, 10, 11, 12, 15, 16, 18, 19, 20, 21)	0.82
			2 “Willingness to be Tested”	*n* = 5 (1, 2, 3, 4, 5)	0.86
			3 “Motivations for testing and role of third parties”	*n* = 10 (6, 7, 8, 13, 14, 17, 22, 23, 24, 25)	0.74
Germany	PRE-ADS-D (Angelidou et al., 2023)	0.78	1 “Concerns about Screening”	*n* = 10 (9, 10, 11, 12, 15, 16, 18, 19, 20, 21)	0.85
			2 “Intention to be Screened”	*n* = 6 (1, 2, 3, 4, 5, 6)	0.87
			3 “Preventive Health Behaviors”	*n* = 9 (7, 8, 13, 14, 17, 22, 23, 24, 25)	0.81
Spain	PRE-ADS (Spanish version)	0.83	1 “Preferences for Preventive AD Testing”	*n* = 5 (1, 2, 3, 4, 5)	0.95
			2 “Mental Health and Emotional Distress”	*n* = 6 (11, 12, 18, 19, 20, 21)	0.83
			3 “Perceived Burden on the family”	*n* = 5 (9, 10, 13, 15, 16)	0.74
			4 “Motivation to Plan the Future”	*n* = 6 (14, 17, 22, 23, 24, 25)	0.80
			5 “Desire to seek further information and counseling from health professionals”	*n* = 3 (6, 7, 8)	0.85
Turkey	PRE-ADS (Turkish version)	0.69	1 “Potential Harms of Screening”	*n* = 10 (9, 10, 11, 12, 15, 16, 18, 19, 20, 21)	0.86
			2 “Acceptance of Screening”	*n* = 8 (1, 2, 3, 4, 5, 6, 7, 8)	0.84
			3 “Motivators for Screening”	*n* = 7 (13, 14, 17, 22, 23, 24, 25)	0.84

^∗^Original validation study. ^∗∗^Cronbach’s α for total scales and factors.

Around the world, family members are the primary caregivers for people with dementia (Brodaty and Donkin, 2009). Caregivers, who are predominantly female (71%), spend about 6 h a day caring for a relative (Alzheimer’s Disease International and Karolinska Institutet, 2018). It is estimated that there are more than 100 million unpaid caregivers in Europe (Kabir et al., 2020). However, cultural differences are to be expected between countries, as there are differences in healthcare systems, as well as in the access or availability of outpatient or inpatient long-term care (Schmachtenberg et al., 2022). Studies have shown that both family members and caregivers have better knowledge about dementia and more positive attitudes toward dementia than the general population (Teichmann et al., 2022). To our knowledge, no studies have examined differences in motivation to perform a pre-symptomatic test between family members and non-family members of people with AD or have focused on cultural differences between European countries. Since the three components that make up “attitude” are cognition (assumptions and beliefs), affect (feelings and emotions), and behavior (actions), it is reasonable to assume that knowledge of dementia through having a family member with AD, as well as the resulting emotional experience, will also influence motivation for a pre-symptomatic screening. Therefore, the aims of this cross-sectional study are: a) to examine the psychometric properties of the PRE-ADS and the subscale “Acceptability of Screening”, b) to compare the attitudes of family members *versus* non-family members toward pre-symptomatic AD screening, c) to compare the attitudes, motivations, perceived benefits and harms between five European countries (Belgium, Germany, Greece, Spain, Turkey) toward pre-symptomatic screening using the translated versions of the PRE-ADS questionnaire.

## Materials and methods

### Study design

This study uses a cross-sectional quantitative design and aims to explore the perspectives of individuals with or without family members with AD regarding pre-symptomatic screening in a diverse European general population, more specifically in five European countries (Belgium, Germany, Greece, Spain, Turkey). The Strengthening the Reporting of Observational Studies in Epidemiology (STROBE) Checklist was used for research reporting (Supplementary Material S2).

### Participants

The samples from the five countries were recruited between April 2021 and June 2023. As the original PRE-ADS questionnaire is in Greek (Makri et al., 2023), the Greek sample was recruited first, from April 2021 to June 2021. After translation and back-translation according to guidelines by Hambleton (2001) into Belgian, German, Spanish, and Turkish, the scales were uploaded on Google Forms, and a convenience sample was gathered through methods such as newsletters, flyer distribution, and social media between May 2022 and June 2023. Inclusion criteria required participants to be over 18 years of age and to have a high level of language proficiency.

### Data collection procedure

The sample of the present study was drawn from five different validation study populations, all of which had validated the questionnaire in their respective country’s general population. Initially, all data were collated into a single SPSS-file (N = 1,298) to detect missing values and obtain the necessary sample size for the current study. Questions with missing values were excluded from the analysis. We created two groups of participants and randomly selected 65 family members of PwD and 65 non-family members from each country’s sample. The final sample size was 650 participants.

### Perceptions regarding pRE-symptomatic Alzheimer’s disease screening (PRE-ADS)–a 25-item questionnaire

The original Greek version of the PRE-ADS questionnaire was developed specifically to measure attitudes, motivations, and barriers to pre-symptomatic screening for AD and is based on the PRISM-PC scale (Boustani et al., 2008) and the Health Belief Model (HBM) (Becker, 1974) (Supplementary Material S2). The scale was validated using a group of university students and informal caregivers of PwD, as described by Makri et al. (2023). The questionnaire covered sociodemographics (age, gender, marital status, education level, occupation), along with information about their past experiences with AD (11 items), followed by the 25 questions of the PRE-ADS scale, scored on a five-point Likert scale, ranging from 1 (strongly disagree) to 5 (strongly agree), resulting in a range of 25–125. A higher score on the questionnaire indicates greater agreement to pre-symptomatic screening for AD, namely, a greater acceptance, greater recognition of the potential benefits associated with pre-symptomatic screening for AD, as well as a greater desire to learn more about AD screening and a lower perception of the potential harms of screening. The ten negatively worded statements in the questionnaire (items 9, 10, 11, 12, 15, 16, 18, 19, 20, 21) were reverse scored for analysis, coding 1 into strongly agreeing and 5 into strongly disagreeing.

### Statistical analysis

All participants (N = 650) were included in the statistical analysis. The data was analyzed using the IBM Statistical Package for Social Sciences (SPSS) Version 28. Descriptive data analysis of the characteristics of the data was described by calculating distributions, mean scores, and standard deviations.

### 25-Item PRE-ADS

Prior to this study, Exploratory Factor Analyses were conducted for each version of the PRE-ADS, resulting in different three-, four- or five-factor models for the scale in each country, depicted in Table 1. The present study assessed the structural validity through Confirmatory Factor Analysis (CFA) to verify the appropriateness of the proposed four-factor solution derived from the original validation study by Makri et al. (2023) for the cross-cultural database. CFA was conducted using IBM SPSS Amos 29 with the maximum likelihood estimation procedure. We assessed model fit using several goodness-of-fit indices, including the chi-square test, comparative fit index (CFI), Tucker-Lewis index (TLI), root mean square error of approximation (RMSEA), and standardized root mean square residual (standardized RMR) (Schreiber et al., 2006; Caballero et al., 2013). Cronbach’s *α* was used for measuring the internal consistency of the scale (Taber, 2018). Pearson correlations among factors were estimated.

### “Acceptability of Screening” subscale

In the validation process of the German PRE-ADS-D (Angelidou et al., 2023), following the approach of Braun et al. (2014), the subscale “Acceptability of Screening” was created, corresponding to factor 2 of the four-factor solution, to draw specific conclusions about the intention to undergo pre-symptomatic AD screening. Five items of the PRE-ADS scale were extracted and converted into a mean score variable, which focuses on assessing participants’ general preference to find out if they are at higher risk for AD (item 1) and their intention to undergo routine testing using different diagnostic methods (items 2, 3, 4, 5). Internal consistency of the subscale was measured by Cronbach’s alpha coefficient. In order to make percentage-based observations about the overall acceptability of pre-symptomatic screening, the mean subscale score was coded into a dichotomized variable. Scores from 1.0 to 3.0 were coded to 0, indicating a rejective attitude toward pre-symptomatic AD screening, and scores over 3.1 to 5.0 were coded to 1, indicating an acceptive attitude toward routine screening (Braun et al., 2014).

### Statistical group-comparisons

All hypotheses are illustrated in Table 2. Independent two-sample t-tests were used to determine whether the total scores of the PRE-ADS and of the subscale “Acceptability of Screening” differed significantly between those participants with and without affected first-degree relatives. The normality of distributions of the outcome variables was confirmed using a combination of visual inspection and checking the values of skewness and kurtosis (George and Mallery, 2010), as formal normality tests are unreliable for large sample sizes (Ghasemi and Zahediasl, 2012; Kim, 2013). In order to investigate possible cross-cultural group differences, first, descriptive comparisons of item response and factor means were used to examine how responses varied across the five countries. Subsequently, it was examined whether subgroups from different European countries differed on the PRE-ADS and “Acceptability of Screening” mean scores. One-way ANOVAs were calculated to test the hypotheses. The reported effect size for ANOVA was Eta-squared (η^2^) (Lakens, 2013). To give an overview of the data and to identify possible confounders, bivariate correlations between different variables were explored using Pearson’s r. Potential confounders were adjusted *post hoc* for all group comparisons by including them in linear regression analyses using the stepwise selection method (Pourhoseingholi et al., 2012). If needed, factorial ANOVAs with confounding variables as fixed factors were rerun to clarify the results of analyses.

**TABLE 2:** Hypotheses of the group comparisons.

Nr	Hypotheses
	Comparisons of family members of PwD *versus* non-family members
(1)	There will be a significant difference in the PRE-ADS mean score between participants with an affected family member with AD and participants without an affected family member
(2)	There will be a significant difference in the “Acceptability of Screening” subscale mean score between participants with an affected family member with AD and participants without an affected family member
	Cross-cultural comparisons
(3)	There will be a significant difference in the PRE-ADS mean score between Belgian, German, Greek, Spanish, and Turkish subgroups
(4)	There will be a significant difference in the “Acceptability of Screening” subscale mean score between Belgian, German, Greek, Spanish, and Turkish subgroups

### Power analysis

We pre-estimated the required sample size *a priori* using G*Power version 3.1.9.7 to ensure that group analyses are not underpowered. The calculation of sample size ran according to the recommendations of Kang (2021) and was based on the research goals and hypotheses of the study. Therefore, the test family “F-tests” with “One-way ANOVA fixed effects” was set with the significance level *α* = 0.05, a medium effect size of d = 0.25, and a power of 1–*β* = 0.95 (Cohen, 1988; Kang, 2021). Power analysis resulted in a required total sample size of N = 305 for all groups combined. Since our database of N = 650 with 130 of each country largely outmatches this threshold, this ensures that the group analyses are not underpowered.

### Ethical considerations

This study was approved by the GAADRD’s Scientific and Ethics Committee in Greece (Meeting Number: 65/06-02–2021), the Ethics Committee of the Faculty for Behavioral and Empirical Cultural Sciences from the Heidelberg University, Germany (AZ Tei 2022 1/2), the Medical Ethics Committee of the Universitair Ziekenhuis (UZ) Brussel and Vrije Universiteit Brussel (VUB), Belgium (EC-2022-264), the Biomedical Research Ethics Committee of Bellvitge University Hospital, Spain (PR253/22), and the Health Sciences Ethics Committee, Izmir University of Economics, Turkey (B.30.2. İEÜSB.0.05.05-20–192). All procedures contributing to this work comply with the ethical standards outlined in the Declaration of Helsinki, which is relevant to the national and institutional committees on human experimentation. Before the survey, informed written consent was obtained from each participant, and they were told that the research was voluntary, confidential, and purely for academic purposes.

## Results

### Participants’ characteristics

Table 3 shows the sociodemographic characteristics of the total sample N = 650 for all five subgroups. The majority of the participants were female (76.5%), highly educated (71.5%), and consisted of adults aged between 20 and 60 years old (73.2%). Half of the participants had a family member with AD, and 26.9% had undergone AD screening, specifically short questionnaires (24.6%), followed by blood sample (9.7%), MRI or PET scan (9.7%), pathological biomarkers (Aβ and tau proteins) (2.3%), or a combination of different diagnostics (53.7%). No missing data were found during descriptive analysis. The sociodemographic characteristics of each country were not evenly distributed because of variations in the sample characteristics of the validation studies. The Belgian subgroup was almost entirely made up of younger adults under the age of 60 (96.2%), while the Spanish and Turkish subgroups have a more even age distribution. The Spanish sample had the highest proportion of male participants (42.3%), the German sample had the highest proportion of participants with lower levels of education (11.5%), and the Turkish subgroup had the most participants with higher levels of education (93.1%). All Greek participants have undergone some type of dementia screening, making up 74.3% of the total 175 screened participants.

**TABLE 3:** Participant characteristics of total sample and countries’ subgroups.

	Full sample		Belgian sample		German sample		Greek sample		Spanish sample		Turkish sample	
Characteristics	*n*	%	*n*	%	*n*	%	*n*	%	*n*	%	*n*	%
Age groups												
20-30	184	28.3	109	83.8	42	32.3	21	11.4	0	0	12	9.2
31-40	75	11.5	8	6.2	20	15.4	18	13.8	7	5.4	22	16.9
41-50	75	11.5	6	4.6	8	6.2	32	24.6	15	11.5	14	10.8
51-60	142	21.8	2	1.5	33	25.4	29	22.3	46	35.4	32	24.6
61-70	111	17.1	2	1.5	17	13.1	24	18.5	27	20.8	41	36.9
71-80	52	8.0	3	2.3	7	5.4	6	4.6	29	22.3	7	5.4
81-90	11	1.7	0	0	3	2.3	0	0	6	4.6	2	1.5
Age (dummy variable)												
Younger adults (< 60)	476	73.2	125	96.2	103	79.2	100	76.9	68	52.3	80	61.5
Older adults (> 61)	174	26.8	5	3.8	27	20.8	30	23.1	62	47.7	50	38.5
Gender												
male	153	23.5	23	17.7	28	21.5	18	13.8	55	42.3	29	22.3
female	497	76.5	107	82.3	102	78.5	112	86.2	75	57.7	101	77.7
Education*												
basic education (9 years or less)	24	3.7	0	0	15	11.5	1	0.8	7	5.4	1	0.8
secondary education (12 years)	155	23.8	56	43.1	32	24.6	26	20	33	25.4	8	6.2
higher education (over 12 years)	471	72.5	74	56.9	83	63.8	103	79.2	90	69.2	121	93.1
Marital status												
divorced	63	9.7	3	2.3	20	15.4	14	10.8	10	7.7	16	12.3
in partnership	94	14.5	43	33.0	18	13.8	18	13.8	9	6.9	6	4.6
single	168	25.8	66	50.8	30	23.1	24	18.5	13	10.0	35	26.9
married	288	44.3	17	13.1	38	29.5	69	53.1	95	73.1	69	53.1
widowed	37	5.7	1	0.8	24	18.5	5	3.8	3	2.3	4	3.1
Affected family member	Yes	No	Yes	No	Yes	No	Yes	No	Yes	No	Yes	No
*n*	325	325	65	65	65	65	65	65	65	65	65	65
Previous Dementia Screening	Yes	No	Yes	No	Yes	No	Yes	No	Yes	No	Yes	No
*n*	175	475	2	128	8	122	130	0	27	103	8	122
%	26.9	73.1	1.5	98.5	6.2	93.8	100	0	20.8	79.2	6.2	93.8

*Note*. total *N* = 650; country subsamples *n* = 130. ∗Participants' education levels were determined by inquiring about their highest degree attained. They were categorized as follows: basic education (participants with either no education, completion of primary school, or completion up to the 9th grade, e.g., Realschulabschluss in Germany), secondary education (participants who completed highschool assigned as 12 years of education, reflecting the standard 12-grade education system) and higher education (participants with any type of completed graduate studies such as a bachelor's degree, master's degree, or PhD/Post-doc).

### 25-Item PRE-ADS

**FIGURE 1:**
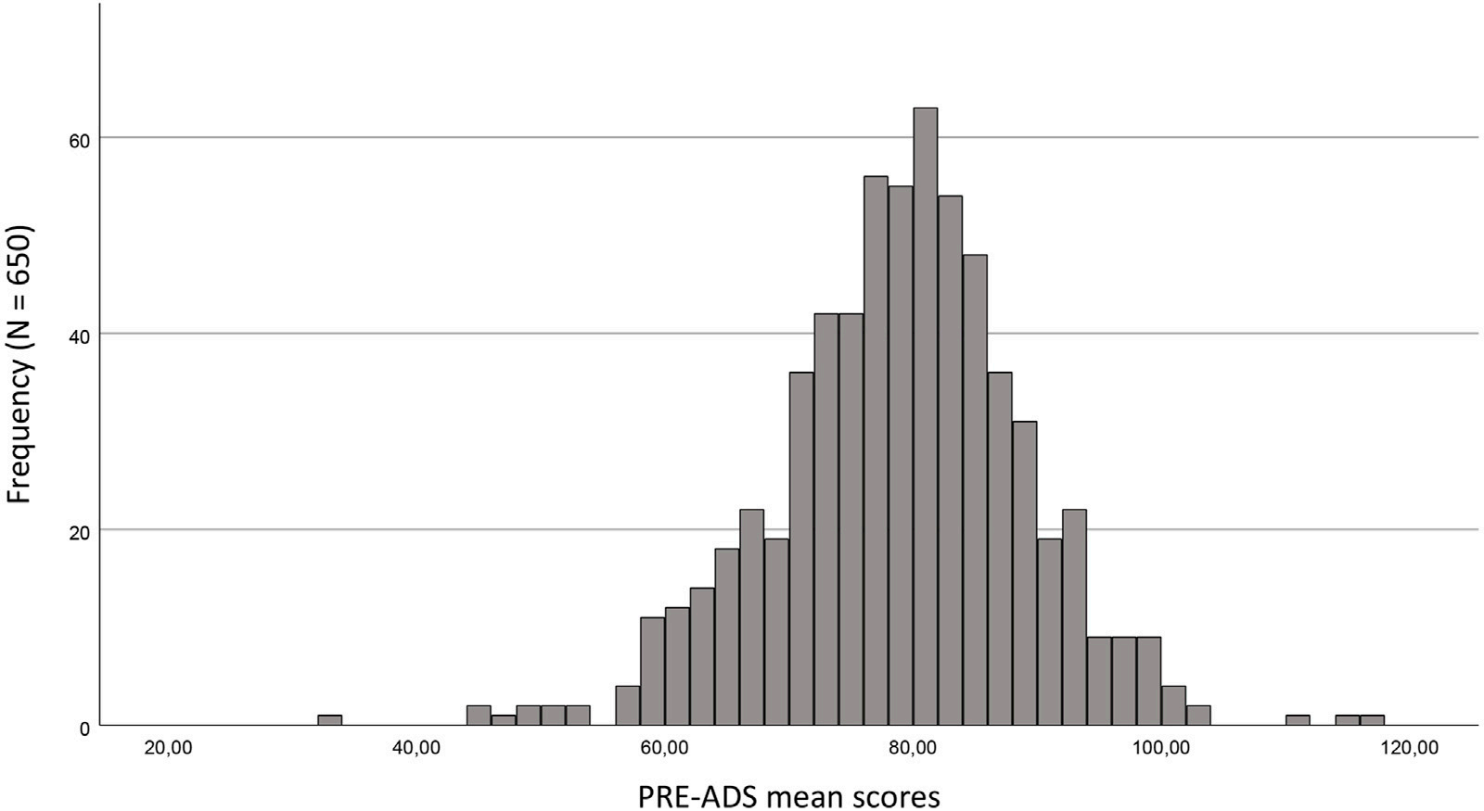
Distribution of PRE-ADS total mean scores (N = 650). PRE-ADS raw total score ranges on a scale from 25–125. Mean score = 78.32. Standard Deviation 10.233. Maximum = 116.0. Minimum = 33.0.

The 25-item PRE-ADS mean score for the total sample (N = 650) was 78.32 (SD = 10.233), and the distribution was slightly negatively skewed (Figure 1), indicating a slightly positive attitude toward pre-symptomatic AD screening. Values of skewness (−0.268) and kurtosis (1.132) are below different thresholds in literature (e.g., < ±2) and indicate a close to normal distribution (George and Mallery, 2010).

### Confirmatory factor analysis

**FIGURE 2:**
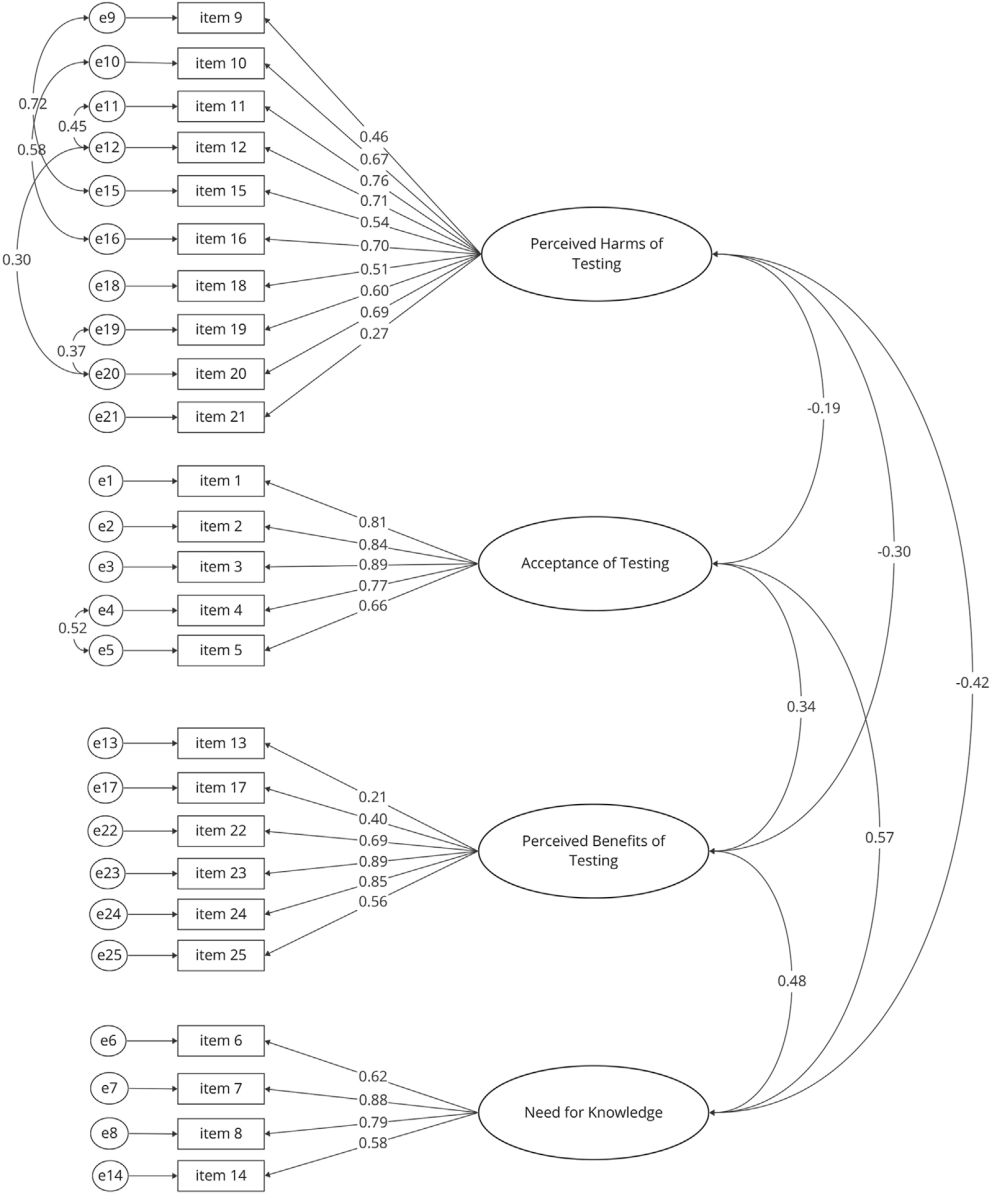
Standardized estimates of the Confirmatory Factor analysis based on the four-factor structure in Makri et al. (2023). This figure displays high error variances of items within the same factors that were selectively included in the model (modification indices >65), factor loadings, and correlations between factors. Correlations between factors are estimated based on the specific overidentified model and differ from the observed (sample) correlations (Pearson) due to model-implied restrictions.

In the Greek PRE-ADS questionnaire, the 25 items represent four hypothesized factors: factor 1 (Perceived Harms of Testing) (items 9, 10, 11, 12, 15, 16, 18, 19, 20, 21), factor 2 (Acceptance of Testing) (items 1, 2, 3, 4, 5), factor 3 (Perceived Benefits of Testing) (items 13, 17, 22, 23, 24, 25) and factor 4 (Need for Knowledge) (items 6, 7, 8, 14). Despite this clear four-factor solution proposed by Makri et al. (2023), the initial CFA model showed a poor fit to the data. The CFI and TLI did not meet the recommended cutoff of >0.90–0.95 as described by different authors (Bentler and Bonett, 1980; Schreiber et al., 2006), while the RMSEA (<0.05) and the standardized RMR (<0.08) were higher than desired (χ^2^ = 2,273,258, df = 268, *p* < 0.001; CFI = 0.780; TLI = 0.754; RMSEA = 0.104; standardized RMR = 0.0809; 90% CI). To improve the model fit, high error variances of items within the same factors (modification indices greater than 65) were selectively included in the model based on theoretical justifications. The revised model is depicted in Figure 2 and showed a reasonable model fit to the data (χ^2^ = 1,034,309; df = 263, *p* < 0.001; CFI = 0.915; TLI = 0.904; RMSEA = 0.065, Standardized RMR = 0.0704, 95% CI). After conducting a more in-depth analysis regarding items with low standardized regression weights (<0.4), squared multiple correlations (<0.2), high residual covariances (>2.0 with many items), and factor loadings (<0.30) (Maydeu-Olivares and Shi, 2017), three items would improve the model fit even more if they were excluded (items 13, 17, 21) (χ^2^ = 701,813; df = 197, *p* < 0.001; CFI = 0.942; TLI = 0.932; RMSEA = 0.061, Standardized RMR = 0.0574, 95% CI). However, because this study aims to explore cross-cultural influences on all 25 items, no item was deleted. All other factor loadings were significant (*p* < 0.001) and ranged from 0.46 to 0.89, except for the aforementioned items, indicating moderate to strong relationships to their respective latent factors.

### Internal consistency

The Cronbach’s alpha value for the PRE-ADS was *α* = 0.76, consisting of the four factors: “Perceived Harms of Testing” (0.86), “Acceptance of Testing” (*α* = 0.90), “Perceived Benefits of Testing” (*α* = 0.77), and “Need for Knowledge” (*α* = 0.79) and indicating a relatively good to excellent level of internal consistency (Taber, 2018). Pearson correlations between factors were all weak to moderate and highly significant, as depicted in Table 4.

**TABLE 4:** Pearson correlations between the factors of the four-factor structure of the PRE-ADS.

Pearson correlations (r)	Factor 1	Factor 2	Factor 3	Factor 4
Factor 1 (Perceived harms of Screening)	-	-		
Factor 2 (Acceptance of Screening)	−0.186^∗∗^	-		
Factor 3 (Perceived Benefits of Screening)	−0.256^∗∗^	0.360^∗∗^	-	
Factor 4 (Need of Knowledge)	−0.327^∗∗^	0.495^∗∗^	0.507^∗∗^	-

Note. total *N* = 650. ^∗∗^ Correlation is significant at the 0.001 level (2-tailed).

### “Acceptability of Screening” subscale

The mean acceptability score for the entire sample was M = 3.2815 (SD = 1.043) with a slightly negatively skewed but close to normal distribution (−0.136) and a kurtosis of −0.612, indicating that the overall tendency of the sample is to have an accepting attitude toward undergoing pre-symptomatic AD screening. Overall, 370 participants (56.9%) expressed positive answers toward the intention to undergo routine pre-symptomatic screening, while 280 (43.1%) indicated that they would not accept routine screening.

### Internal consistency

Internal consistency turned out to be excellent for the mini-scale (*α* = 0.90), which ensures its reliability and confirms the one-dimensionality of the scale.

### Statistical group-comparisons

To give an overview of the data, bivariate Pearson’s correlations between different data variables were explored. The PRE-ADS and its subscale “Acceptability of Screening” revealed weak positive correlations with having an affected family member (r = 0.152, r = 0.203), the country of origin (r = −0.183, r = −0.297), and previous dementia screening (r = 0.236, r = 0.290), all highly significant (*p* > 0.001). Additionally, the subscale had a weak positive correlation with the age of the participants (r = 0.095, *p* > 0.05). The scales showed a strong positive correlation to each other (r = 0.687, *p* < 0.001). Demographic variables (age, gender, education level, marital status) along with variables with significant correlations were included in *post hoc* multiple linear regression models to adjust for possible confounders in our group comparisons (Pourhoseingholi et al., 2012). Identified confounders were included as fixed factors in the analyses.

### Attitudes of family members of PwD *versus* non-family members toward pre-symptomatic AD-screening

Statistical results of the known-group tests are presented in Table 5. The PRE-ADS mean score was statistically different between participants, with and without affected family members (t (648) = 3.914, *p* < 0.001), showing a small to moderate effect size (Cohen’s d = 0.307). Similarly, for the subscale “Acceptability of Screening”, analyses revealed significant differences in mean score between participants with an affected family member and those without such experience (t (648) = 5.290, *p* < 0.001) with a moderate effect (Cohen’s d = 0.415). After adjusting for potential confounding variables in *post hoc* regression analyses, the effect of “affected family member” remained statistically significant. No changes in the Beta-Coefficient were observed after including possible confounders, indicating a relatively clear relationship to both mean scores (Table 6). This suggests that individuals with affected family members tend to score slightly higher than those without affected family members in both the whole PRE-ADS scale as well as on the subscale “Acceptability of Screening” that only assesses the overall intention to be screened.

**TABLE 5:** Group-comparisons between participants with and without family members with PwD.

Group variable	Affected family members				
Independent test	Yes (n = 325)	No (n = 325)	T^1^	d^2^	P^3^
PRE-ADS mean source (SD) [range 25.0–125.0]	78.880 (10.821)	76.722 (9.371)	3.914	0.307	<0.0001
“Acceptability of Screening” mean score (SD)[range 1–5]	3.494 (1.057)	3.069 (0.984)	5.290	0.415	<0.0001

*Note.* Total *N* = 650. ^1^T = T-test statistic. ^2^d = Cohen’s d effect size. ^3^p = significance.

**TABLE 6:** Regression models for the PRE-ADS and the “Acceptability of Screening” subscale.

PRE-ADS	*β*	SE	95% CI		*p*
Variables			LL	UL	
Affected family member	0.160	0.773	1.759	4.793	**< 0.001**
Country of Origin	−0.064	0.375	−1.200	0.271	0.215
Previous Dementia Screening	0.194	1.182	2.161	6.803	**< 0.001**
Education level	−0.085	0.731	−3.058	-0.187	**0.027**

Model	R² 7	SE			*p*
	0.087	9.809			**< 0.001**

“Acceptability of Screening” subscale	*β*	SE	95% CI		*p*
Variables			LL	UL	
Affected family member	0.212	0.075	0.294	0.590	**< 0.001**
Country of Origin	−0.249	0.038	−0.259	-0.108	**< 0.001**
Previous Dementia Screening	0.130	0.117	0.076	0.535	**0.009**
Education level	−0.114	0.071	−0.361	−0.081	**0.002**
Marital status	0.088	0.028	0.010	0.119	**0.020**

Model	R²				*p*
	0.164	0.957			**< 0.001**

*Note*. total *N* = 650. ^1^β = Standardized Beta-Coefficients. ^2^SE = Standardized Error. ^3^CI= Confidence Interval. ^4^LL = Lower Limit. ^5^UL = Upper Limit. ^6^p = significance. ^7^R² = R Square, coefficient of determination in regression analysis. Additional predictors age, gender as well as marital status for the PRE-ADS were automatically excluded during analysis.

### Cross-cultural differences in attitudes toward pre-symptomatic AD screening

Descriptive item and factor mean score level comparison Supplementary Table S1 shows the mean item responses and factor scores for each of the different country samples. Factor 1 includes item 10 (My family will suffer emotionally), which has the lowest response score overall (M = 1.75 (SD = 0.96)) as well as for the different subgroups, ranging from 1.51 (SD = .78) in the Spanish subgroup to 2.15 (SD = 1.44) in the Turkish subgroup. Items within this factor include the perceived family burden, emotional distress, mental pain, and anxiety, with cores closer to 1 demonstrate agreement with the potential harms of screening, whereas scores closer to 5 indicate disagreement. The factor “Need for Knowledge” ranked highest among factors and includes item 14, being informed about new advancements in AD treatment and prevention, which holds the highest mean score for item responses overall 4.21 (SD = 0.89).

### Statistical cross-cultural group-comparisons

**FIGURE 3:**
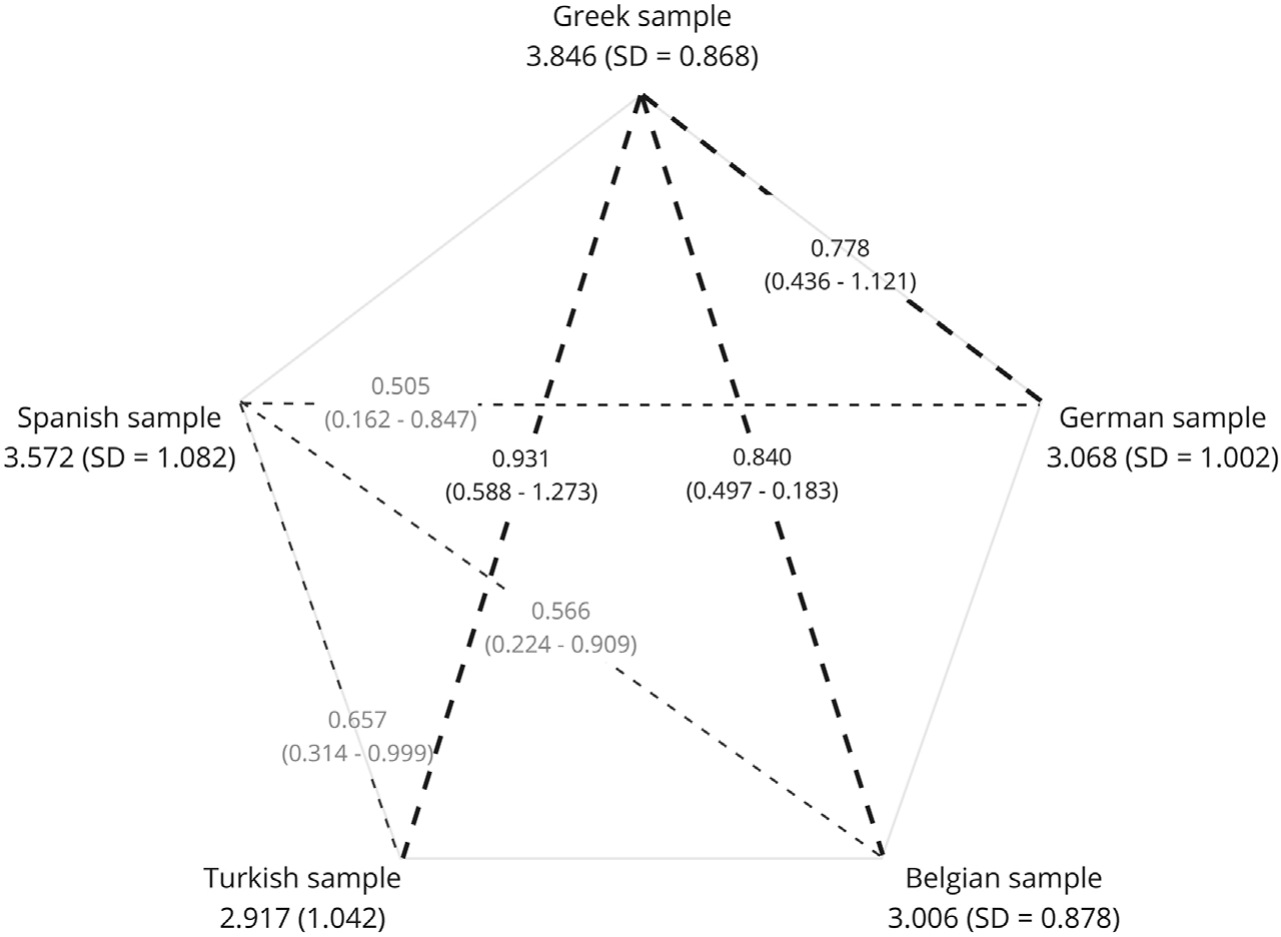
Pairwise comparisons of the “Acceptability of Screening” Subscale´s mean scores. Mean scores and standard deviations are marked under each country. Subsamples with significant differences in their mean scores are connected with dashed lines. Thicker dashed lines represent pairwise comparisons related to Greece, while thinner dashed lines correspond to comparisons involving Spain. The dashed lines are annotated with mean differences and the corresponding 95% confidence intervals, providing insights into the magnitude and precision of the observed variations. A standard error of 0.122 is consistent across all pairwise comparisons.

Table 7 illustrates the results of the group comparisons, while *post hoc* regression analyses to adjust possible confounders are shown in Table 6. Initial ANOVA showed significant differences between groups in the mean score of PRE-ADS across different countries of origin (F (4) = 11.438, η^2^ = 0.066, *p* < 0.001). However, in the *post hoc* regression analysis, when adjusting the confounding variables, the significance and predictive value diminished (*β* = -0.064, *p* > 0.05). Specifically, education level, marital status, and whether participants had already undergone any kind of dementia screening seemed to have a greater effect on the PRE-ADS mean score. When including these confounders in the analysis, factorial ANOVA showed no significant difference across groups. In the case of the “Acceptability of Screening” mean score, the different subsamples also yielded significantly different mean scores among the five countries subsamples initially (F (4) = 22.284, η^2^ = 0.121, *p* < 0.001). The study country remained a significant predictor even after adjusting for education level, marital status, and previous dementia screening in the regression model (*β* = −0.249, *p* < 0.001). After including the aforementioned confounders in a factorial ANOVA, the clarified effect of “Country of Origin” was relatively small, suggesting that the group differences among countries are modest yet significant (F (4) = 2.875, partial η^2^ = 0.02, *p* < 0.05). Pairwise comparisons revealed that the Greek population had a significantly higher total score when compared to all other countries, followed by the Spanish participants, showing significantly higher scores than Germany, Belgium, and Turkey, which had the lowest scores among all subgroups (Figure 3).

**TABLE 7:** Results of the cross-cultural group comparisons.

Initial Analysis of Variance (ANOVA)	Belgian sample	German sample	Greek sample	Spanish sample	Turkish sample	F^1^	η² ^2^	P^3^
PRE-ADS mean score (SD)(range 25.0 – 125.0)	77.223(8.430)	77.285(9.747)	82.923(9.457)	79.123(10.019)	75.077(11.664)	11.438	0.066	< 0.001
“Acceptability of Screening”mean score (SD) (range: 1-5)	3.0062(0.878)	3.0677(1.002)	3.8462(0.868)	3.5723(1.082)	2.9171(1.042)	22.284	0.121	< 0.001

Factorial ANOVA^4^	Belgian sample	German sample	Greek sample	Spanish sample	Turkish sample	F	Partial η2 ^5^	p
PRE-ADS mean score (SD)(range 25.0 – 125.0)	77.223(8.430)	77.285(9.747)	82.923(9.457)	79.123(10.019)	75.077(11.664)	2.358	0.016	> 0.05
“Acceptability of Screening”mean score (SD) (range: 1-5)	3.006(0.878)	3.068(1.002)	3.846(0.868)	3.572(1.082)	2.917(1.042)	2.875	0.020	< 0.05

*Note*. total *N* = 650; country subsamples *n* = 130. ^1^F = F-statistic for ANOVA. ^2^η_2_ = Eta-squared, effect size for ANOVA. ^3^p = significance. ^4^Factorial ANOVA = ANOVA with multiple independent variables to reveal corrected group differences after adjusting for confounders. Confounders are education level, marital status, previous dementia screening. ^5^Partial η_2_ = Partial Eta-squared, effect size for ANOVA with multiple factors (independent variables).

## Discussion

The aim of this study was to investigate the perspectives of individuals, both with and without family members diagnosed with AD, regarding pre-symptomatic screening across five different European countries: Belgium, Germany, Greece, Spain, and Turkey. The four-factorial structure of the Greek version of the PRE-ADS (Makri et al., 2023) could be confirmed. Both the 25-item scale and its four factors (“Perceived Harms of Testing”, “Acceptance of Testing”, “Perceived Benefits of Testing”, and “Need for Knowledge”) showed acceptable to good internal consistency. Overall, 56.9% of the total sample expressed a willingness to pre-symptomatic AD screening. Known group analyses showed significant differences between participants with and without an affected family member. An international comparison revealed modest cross-cultural differences in the intention to be screened (“Acceptability of Screening” subscale) across the subgroups of the five European countries but not in the whole scale’s mean score after adjusting for confounding variables.

### Psychometric properties

#### 25-Item PRE-ADS

Internal consistency of the PRE-ADS Scale is in line with the previous validation studies such as the prototypical Greek version of the PRE-ADS (Makri et al., 2023), the German version (Angelidou et al., 2023), and the other three translations (Table 1). Similar scales that focus on dementia screening also reveal comparable *α* levels (Galvin et al., 2006; Boustani et al., 2008; Holsinger et al., 2011; Wikler et al., 2013; Braun et al., 2014; Gooblar et al., 2015).

Inter-factor correlations were weak to moderate (Table 4), and the strongest correlation emerging between factor 2 (“Acceptance of Testing”) and factor 4 (“Need for Knowledge”). Participants with a positive attitude toward pre-symptomatic AD screening, therefore, also expressed a stronger desire for knowledge, which underlines the importance of facilitating access to comprehensive information for those considering such testing. It also highlights the significance of genetic counseling by a professional and aligns with the fundamental principles of genetic counseling, where providing clear, unbiased information is key to empower individuals with the understanding that they need to give informed consent and make independent choices about whether to pursue genetic testing (Rink and Kuller, 2018). The positive correlation between factor 2 and factor 3 (“Perceived Benefits of Testing”) is in line with the previous validation study of Angelidou et al. (2023), who also found a comparable correlation between their second (“Intention to be Screened”) and third factor (“Preventive Health Behaviors”). The relationship between both constructs is underlined by other studies, as the majority of the mentioned benefits are the most convincing motivations for pursuing pre-symptomatic screening (Chao et al., 2008; Wikler et al., 2013; Rolf et al., 2021). Conversely, factor 1, “Perceived Harms of Testing”, exhibits weak negative correlations with the other factors. In practical terms, when individuals perceive potential harms as a significant concern, their agreement on the Likert scale diminishes regarding factors related to the benefits of testing, acceptance of testing, and the need for knowledge. The harms of testing included in this questionnaire are known risks for pre-symptomatic screening, aside from other known harms including over-diagnosis, internalized and public stigma as well as political risks about voting, driving, or workplace discrimination on the basis of dementia risk (Kang et al., 1987; Moscarillo et al., 2007; Smedinga et al., 2018).

Structural validity for the PRE-ADS was confirmed, as the proposed four-factor structure by Makri et al. (2023) is also acceptable for this multicultural dataset. While the model’s fit was poor initially, it significantly improved after incorporating high error variances of items within the same factors. Although the TLI and CFI still fell slightly below the threshold of 0.95 (Schreiber et al., 2006), many researchers consider a CFI and TLI above 0.90 as indicative of a reasonable fit (Bentler and Bonett, 1980; Byrne, 1994; Hu and Bentler, 1999; Caballero et al., 2013). As depicted in Figure 2, high error variances were mainly identified within factor 1, “Perceived Harms of Testing”, possibly because this factor contains a substantial number of items that share similarities in wording and content, which can result in shared variance between items. The removal of items 13, 17, and 21 would raise the model’s fit. A possible explanation for item 13 (“I would improve my quality of life”) having low values could be due to similarity with item 25 (“I would be motivated to have a healthier lifestyle”). This item was also problematic in the validation study of the PRE-ADS as it revealed a low factor loading and cross-loadings on more than one factor (Angelidou et al., 2023). Item 21 (“I would give up on life”) could show low scores because participants generally disagreed with this item, possibly due to its vague yet intense nature, so that the responses to this item contrast significantly with the other negatively worded statements (Supplementary Table S1). Finally, item 17 (“My family would have a better chance to take care of me”) was also problematic in the German validation study (Angelidou et al., 2023) as it would raise Cronbach’s *α* if deleted. On the other hand, it was a valid item for the Greek validation study (Makri et al., 2023), indicating that family as a resource and its role in care might be influenced by cultural or healthcare differences of different countries. Because of this study’s objectives, however, we decided not to remove any of the 25 items. In future studies, it is important to consider the wording and content carefully and to remove redundant items to enhance the psychometric properties of the PRE-ADS.

The PRE-ADS assesses various constructs of pre-symptomatic AD screening. However, as it combines numerous topics, drawing specific conclusions about significant differences would be rather vague. Therefore, it might prove beneficial to divide the 25 items into four specific subscales based on factor structure (harms, benefits, acceptance, need for knowledge). Additionally, it may also be advantageous to ask questions on the benefits and harms of screening before asking about an individual’s preference to undergo screening. This change in item order was already proposed in the German validation paper (Angelidou et al., 2023), as it offers respondents valuable context to assess whether the potential benefits outweigh the potential risks of screening (Smedinga et al., 2018) so they can have a balanced decision-making process for the questions about the acceptance of screening.

#### “Acceptability of Screening” subscale

The subscale demonstrated excellent internal consistency. It is identical to factor 2 (“Acceptance of Testing”) of the original validation study (Makri et al., 2023) and captures the willingness to undergo pre-symptomatic dementia screening by asking the first five questions of the PRE-ADS. Overall acceptance for the present European-mixed sample was 56.9%, which is higher than the acceptability rate of 51.2% of the German general population sample used in Angelidou et al. (2023), indicating differences in sample characteristics. Both results are lower than the rates found in similar studies. However, it is important to underline differences in sample characteristics as well as the fact that the other studies focus on diagnostic dementia screening instead of predictive pre-symptomatic testing (Holsinger et al., 2011; Fowler et al., 2012; Braun et al., 2014). The subscale “Acceptability of Screening” could play an essential role in clinical practice in the future, as it is practical to disseminate and quickly assess the acceptance of pre-symptomatic AD screening, enhancing informed consent and individual decision making of someone considering genetic screening.

### Statistical group-comparisons

#### Attitudes of family members of PwD *versus* non-family members toward pre-symptomatic AD-screening

One of the study’s objectives was to compare the attitudes of people with and without an affected family member toward pre-symptomatic AD screening. As Table 8 illustrates, hypotheses 1 and 2 were confirmed in the present study. The higher mean score on the PRE-ADS in people with affected family members could be influenced by several factors. Studies about family members of PwD have shown that their direct personal experience influences their perception and knowledge about the disease (Teichmann et al., 2022). As described by Alpinar-Sencan et al. (2020), who compared the attitudes toward genetic testing for APOE4 between family caregivers and laypersons in a qualitative setting, the increased awareness and experience of family members were interlinked to their attitudes toward testing. In fact, the authors came to the conclusion that, while their core intention to undergo screening may not differ substantially, the reasoning and the quality of arguments of family members of PwD showed important differences in relation to the laypersons. In the present study, family members of PwD had a significantly higher PRE-ADS score, which may indicate that compared to the control group, they may be more likely to consider pre-symptomatic AD screening by showing a greater recognition of the potential benefits rather than of potential harms and a greater desire to learn more about pre-symptomatic AD screening. Similarly, participants with affected family members also revealed higher mean scores in the subscale “Acceptability of Screening”. These results are comparable to the German sample in the validation study of the PRE-ADS-D (Angelidou et al., 2023), indicating that in both populations, the German and the international sample, people with family history have a stronger willingness to find out about their risk of developing AD in the future. A similar study has also pointed out the importance of a positive family history and the intention to undergo pre-symptomatic AD screening (Caselli et al., 2014). One explanation could be that family members of persons with AD have an increased knowledge about AD (Melchior and Teichmann, 2023). In a Japanese cross-sectional study, a higher level of dementia literacy among 854 older adults appeared to increase willingness to screen for dementia, which could potentially extend to pre-symptomatic screening (Aihara and Maeda, 2020). However, a Belgian study involving students found that greater knowledge about AD actually led to less willingness to undergo predictive testing for AD. This effect was attributed to the later onset and greater variability in the age of onset of AD (Welkenhuysen et al., 1997), which complicates planning for the future, undermining its perceived benefits in the context of predictive AD testing (Decruyenaere et al., 1993; Tibben et al., 1997). Another explanation for our results could be that family members of PwD perceive themselves to be at increased genetic risk of developing dementia. Indeed, additional analyses by Angelidou et al. (2023) revealed a significant association between a positive family history and concern about developing dementia. The perceived susceptibility could lead to a stronger intention to undergo pre-symptomatic screening in order to better understand their own genetic risk profile. In fact, in the Alpinar-Sencan et al. (2020) study, participants with a family history were more likely to recognize certain benefits of early detection through screening, such as the possibility of starting medication early to slow the disease. However, these benefits become questionable, especially in the case of LOAD, firstly because of its late onset and secondly due to the diagnostic uncertainty of pre-symptomatic AD screening (Bunnik et al., 2018). Further, a positive family history does not always increase willingness to undergo screening. Alanazy et al. (2019), who were the first to examine public perceptions of pre-symptomatic dementia testing in Saudi Arabia, found that those with a positive family history were less likely to undergo such screening. The authors explained this result by the potential perceived emotional and psychological distress upon realizing that they could suffer a similar deterioration in their health as their loved ones. This highlights another ethical issue that genetic data always include family data, and that family implications, such as the right of family members not to know, must be treated with respect and care (Forbes Shepherd et al., 2017). In summary, the impact of positive family history on the willingness to undergo pre-symptomatic or predictive AD screening is complex, with mixed findings across different studies and populations. Further research is needed, as the discrepancy in results between the aforementioned studies might result due to sampling differences in age, religion, culture, and values of the studied populations.

**TABLE 8:** Overview of all hypotheses and results.

Nr.	Hypothesis	PRE-ADS	Subscale^1^
(1, 2)	There will be a significant difference in the […] mean score between participants with an affected family member with AD and participants without an affected family member.	✔	✘
(3, 4)	There will be a significant difference in the […] mean score between Belgian, German, Greek, Spanish and Turkish subgroups.	✔	✔

^1^“Acceptability of Screening” subscale.

#### Cross-cultural differences in the “Acceptability of Screening” subscale

Looking at the results, the Greek subgroup “Acceptability of Screening” score is significantly higher when compared to all other countries, especially Turkey, Belgium, and Germany. Greek participants seem to have a high interest and more positive attitudes toward the benefits of pre-symptomatic screening as they have the highest scores in factors 3 and 4 (Supplementary Table S1). These findings could stem from a variety of socio-cultural and healthcare-related factors unique to Greece. For example, the Greek healthcare system tends to have an emphasis on early detection and preventive measures (Myloneros and Sakellariou, 2021; Kampouraki et al., 2023), which could promote positive perceptions about the benefits of pre-symptomatic AD screening in healthcare professionals as well as the general population. However, the differences between the Greek sample and the others may result from unique sample characteristics. The majority of the Greek participants were recruited through the Panhellenic Federation of Alzheimer’s Disease and Related Disorders, which has actively provided various services, including awareness campaigns and educational events for the general public and families dealing with AD (Karagiozi et al., 2017; Tsolaki et al., 2021). Consequently, a significant number of Greek families were aware of screening programs. As a result, all Greek participants had already undergone some form of dementia screening. This probably resulted in selection bias of the Greek sample, which was adjusted for in the analyses but must still be considered when interpreting the findings. The Spanish subgroup also showed an interest in pre-symptomatic AD screening, as evidenced by their higher scores in the “Acceptability of Screening” Subscale. This may be linked to the family-oriented cultures of the Southern European countries, such as Spain and Greece, and their strong tradition of family caregiving (Calvó-Perxas et al., 2018; Werner et al., 2019; Koukouli et al., 2022). Individuals from these societies might, therefore, be more receptive to early diagnosis, hoping to have more time to prepare. They may also tend to be aware of the harms associated with dementia care, including costs and family burden. Additionally for Spain, authors report issues in the healthcare system, which lacks a well-structured plan for AD patients, their family members, and informal caregivers (Turró-Garriga et al., 2021). This can be observed in the lower scores of factor 1 (“Perceived Harms of Testing”), implying that the Greek and Spanish populations were particularly concerned about the harms of pre-symptomatic screening. The Turkish subgroup had the lowest scores overall in our analyses, implying a generally lower interest in pre-symptomatic AD screening. A variety of religious, socio-cultural, and healthcare-related factors could explain these differences to an extent. For example, cultural norms like filial piety are deeply enrooted in societies of Eastern Islamic cultures, therefore, caring for older family members is a culturally appreciated and self-evident practice. Additionally, there is a general dissatisfaction in Turkish healthcare services and nursing homes. For families and society, this might provide an intrinsic motivation, preparedness, as well as self-evidence for elderly care provision (Kara, 2007; Ar and Karanci, 2019). This can impact an individual’s perception of the need for predictive screening, as they may believe their family will provide care regardless of a diagnosis. Another potential explanation for the low interest in screening may be a lack of dementia knowledge among healthcare professionals and society. In a study conducted in Turkey (Öz et al., 2022), involving 1,551 persons from 53 cities, about half of the participants considered dementia as a natural consequence of aging. This limited knowledge about dementia might subsequently lead to limited awareness about pre-symptomatic AD screening or genetic testing in general. The samples from the Central European countries Belgium and Germany fall in the lower middle of cross-cultural comparisons and show no significant differences from each other, indicating similar cultural or healthcare-related influences. When it comes to dementia, both have well-established healthcare systems and offer a substantial number of healthcare facilities, caregiver training, and support groups. Medical services related to dementia care are generally covered, and, additionally for Germany, familial caregivers are entitled to receive respite care payment (Alzheimer Europe, 2013). The Central European countries also have stronger cultural norms about privacy and data protection and stricter guidelines regarding genetic testing, including Germany’s Gendiagnostikgesetz (GEKO, 2011). Moreover, since the predictive value of pre-symptomatic testing for APOE4 remains to date very questionable, medical associations like the German Association for Psychiatry, Psychotherapy, Psychosomatics, and Neurology strictly advise against it (DGPPN, 2017). Given these implications, pre-symptomatic screening for AD might appear less favorable or attractive for residents of Germany and Belgium, which is a possible explanation for our results. These findings highlight that attitudes toward pre-symptomatic AD screening could be influenced by personal experience and cultural backgrounds. As the demand and availability of pre-symptomatic screening in general rises, cross-cultural investigations can contribute to the implementation of European counseling programs and other healthcare services that incorporate the cultural needs of each specific nation (Justiss et al., 2009; Makri et al., 2023). While some theoretically based explanations were discussed in this paper, further research is needed to explore the reasons for cross-cultural differences in attitudes toward pre-symptomatic AD screening.

### Strengths and limitations

This is the first study that enables an unprecedented European cross-cultural comparison of attitudes regarding pre-symptomatic AD screening with a substantial sample size of 650, encompassing five distinct nations—Belgium, Germany, Greece, Spain, and Turkey. The study has a unique strength in that it incorporates the perspectives of researchers from each of the five countries involved with an insider’s understanding of the cultural, social, and healthcare nuances of their respective nations. Several limitations in the sample should be considered when interpreting our findings. As previously mentioned, there are unevenly distributed sample characteristics across the different countries’ subsamples, resulting from the different recruitment methods of the previous validation studies. The most important one is that the Greek participants have undergone previous dementia screening, which might result in selection bias, as they might stand favorably toward pre-symptomatic AD screening. Second, subsamples may not be representative for entire countries, e.g., the Belgian sample mostly included students of young age or the proportion of marital status and education level across the different countries’ subsamples and overall. While these issues were accounted for in the analysis, the results and their generalizability have to be interpreted with caution. Further research studies with larger samples and controlled recruitment strategies should be conducted to further explore cross-cultural differences in attitudes toward pre-symptomatic AD screening in Europe.

## Conclusion

The present study aimed to investigate attitudes, motivations, and harms related to pre-symptomatic AD screening among individuals with and without affected family members within a multicultural sample encompassing participants from five European countries: Belgium, Germany, Greece, Spain, and Turkey. The study findings demonstrate that the PRE-ADS tool, along with its “Acceptability of Screening” subscale, is a reliable and valid instrument for assessing these attitudes. More than half of the diverse European sample expressed a favorable disposition toward pre-symptomatic AD screening. Personal experience with an affected family member emerged as a significant factor motivating a more positive attitude toward pre-symptomatic screening. To gain deeper insights into these relationships, future research should delve into the connections between family history, knowledge about dementia, attitudes toward pre-symptomatic AD screening, and comprehension of the benefits and limitations of such screenings. This study identified possible cross-cultural differences across European countries in attitudes toward pre-symptomatic AD screening, which may be due to cultural and religious differences, as well as differences in healthcare and healthcare attitudes, and need to be investigated in the future.

## Data Availability

The raw data supporting the conclusion of this article will be made available by the authors without undue reservation.

## Abbreviations

AD, Alzheimer’s disease; APP, amyloid precursor protein; CFA, confirmatory factor analysis; CFI, comparative fit index; CSF, cerebrospinal fluid; FAD, familial Alzheimer’s disease; GAADRD, greek association of alzheimer disease and related disorders; GECONEU, genetic counseling in european universities; GWAS, genome-wide association studies; NAR, network aging research; LOAD, late-onset AD; NFTs, neurofibrillary tangles; PCA, principal component analysis; PRE-ADS, perceptions regarding pRE-symptomatic alzheimer’s disease screening; PRIM-PC, perceptions regarding investigational screening for memory in primary care; PwD, people with dementia; RMR, root mean square residual; SPSS, statistical package for social sciences; TLI, tucker-lewis index; UZ, universitair ziekenhuis; VUB, vrije universiteit brussel.
