# Supplementary material for: Attitudes toward pre-symptomatic screening for Alzheimer’s dementia in five European countries: a comparison of family members of people with Alzheimer’s dementia versus non-family members
Source: Front Genet. 2023 Dec 15;14:1305107. doi: 10.3389/fgene.2023.1305107 (PMC10757380; doi:10.3389/fgene.2023.1305107)
Supplement: Supplementary file 1 [file Table1.DOCX]

Supplementary Material

Attitudes toward pre-symptomatic screening for Alzheimer’s Dementia in five European countries: a comparison of family members of people with Alzheimer’s Dementia versus non-family members

#### Ioanna Antigoni Angelidou, Marina Makri, Konrad Beyreuther, Mercè Boada, Akyllina Despoti, Sebastiaan Engelborghs, Andrea Miguel, Isabel Rodríguez, Hannah Stocker, Joke Temmerman, Magdalini Tsolaki, Görsev Yener, Deniz Yerlikaya, Birgit Teichmann^*^

*** Correspondence:** Dr. Birgit Teichmann: teichmann@nar.uni-heidelberg.de

**Supplementary material 1**

**Supplementary Table 1.** Means of item and factor responses of the PRE-ADS across subsamples

| **Groups** | | | | | | | |
| --- | --- | --- | --- | --- | --- | --- | --- |
|  | | **Total** | **Belgium** | **Germany** | **Greece** | **Spain** | **Turkey** |
| **Factors and items* Factor and item mean score (SD)** | | | | | | | |
| **Factor 1 Perceived harms of testing** | | **2.42 (0.68)** | **2.52 (0.56)** | **2.45 (0.61)** | **2.26 (0.66)** | **2.23 (0.53)** | **2.64 (0.88)** |
| **Nr.** | **Item** |  |  |  |  |  |  |
| 9. ** | My family will suffer from the additional costs of my care. | 2.47 (1.22) | 2.93 (1.14) | 2.58 (1.17) | 2.13 (1.01) | 1.93 (1.04) | 2.79 (1.40) |
| 10.** | My family will suffer emotionally. | 1.75 (0.96) | 1.74 (0.60) | 1.59 (0.81) | 1.75 (0.84) | 1.51 (0.78) | 2.15 (1.44) |
| 11.** | I feel that I would be overwhelmed by mental pain. | 2.32 (1.02) | 2.35 (0.99) | 2.55 (0.96) | 2.19 (0.98) | 2.15 (0.92) | 2.38 (1.18) |
| 12.** | I feel that I would be overwhelmed by intense anxiety. | 2.29 (0.99) | 2.15 (0.91) | 2.38 (0.95) | 2.12 (0.91) | 2.26 (0.92) | 2.55 (1.19) |
| 15.** | My family would suffer financially. | 2.58 (1.17) | 3.02 (1.04) | 2.78 (1.07) | 2.31 (0.96) | 1.95 (0.98) | 2.82 (1.42) |
| 16.** | My family would suffer emotionally. | 1.88 (0.95) | 1.78 (0.61) | 1.85 (0.83) | 1.93 (0.87) | 1.59 (0.81) | 2.19 (1.36) |
| 18.** | I think that others will treat me in a different way. | 2.37 (0.97) | 2.32 (0.88) | 2.25 (0.88) | 2.27 (0.80) | 2.28 (0.89) | 2.75 (1.25) |
| 19.** | I would be depressed. | 2.53 (0.97) | 2.85 (0.86) | 2.31 (0.95) | 2.28 (0.93) | 2.49 (0.78) | 2.71 (1.16) |
| 20.** | I would be anxious. | 2.35 (0.95) | 2.29 (0.85) | 2.30 (0.99) | 2.11 (0.84) | 2.55 (0.83) | 2.52 (1.16) |
| 21.** | I would give up on life. | 3.70 (0.98) | 3.82 (0.96) | 3.92 (0.81) | 3.53 (0.88) | 3.65 (0.95) | 3.60 (1.20) |
| **Factor 2 Acceptance of testing (“Acceptability of Screening”-Subscale)** | | **3.28 (1.04)** | **3.01 (0.88)** | **3.06 (1.02)** | **3.85 (0.87)** | **3.57 (1.08)** | **2.91 (1.04)** |
| 1. | I would like to know if I am at higher risk than others for developing Alzheimer’s disease. | 3.70 (1.13) | 3.59 (0.95) | 3.51 (1.20) | 4.18 (0.97) | 3.95 (1.06) | 3.25 (1.21) |
| 2. | I would like to be tested for the presence of AD on a regular basis with a short questionnaire. | 3.48 (1.24) | 3.13 (1.14) | 3.34 (1.22) | 4.16 (0.90) | 3.62 (1.18) | 3.13 (1.41) |
| 3. | I would like to be tested for the presence of AD on a regular basis with a blood sample. | 3.37 (1.24) | 3.07 (1.07) | 3.15 (1.28) | 4.00 (1.01) | 3.58 (1.20) | 3.06 (1.36) |
| 4. | I would like to be tested for the presence of AD on a regular basis with pictures of my head or brain (CT-scan or MRI). | 3.04 (1.26) | 2.69 (1.11) | 2.71 (1.18) | 3.56 (1.13) | 3.57 (1.22) | 2.68 (1.29) |
| 5. | I would like to be tested for the presence of AD on a regular basis with the use of biomarkers in cerebrospinal fluid (Aβ amyloid, t-protein). | 2.82 (1.23) | 2.55 (1.10) | 2.63 (1.11) | 3.32 (1.19) | 3.14 (1.18) | 2.46 (1.35) |
| **Factor 3 Perceived benefits of testing** | | **3.54 (0.69)** | **3.45 (0.59)** | **3.48 (0.67)** | **3.84 (0.51)** | **3.66 (0.59)** | **3.25 (0.88)** |
| 13. | I would improve my quality of life. | 3.56 (1.07) | 3.55 (0.87) | 3.56 (0.96) | 3.89 (0.83) | 3.05 (1.12) | 3.77 (1.12) |
| 17. | My family would have a better chance to take care of me. | 3.28 (0.97) | 3.31 (0.79) | 3.02 (0.88) | 3.75 (0.79) | 3.48 (0.92) | 2.81 (1.15) |
| 22. | I would have more time to plan my future. | 3.27 (1.03) | 3.05 (0.93) | 3.16 (1.02) | 3.49 (0.80) | 3.59 (0.95) | 3.04 (1.28) |
| 23. | I would have more time to talk with my family about my health care. | 3.65 (0.96) | 3.70 (0.89) | 3.66 (0.95) | 3.81 (0.70) | 3.85 (0.85) | 3.22 (1.20) |
| 24. | I would have more time to talk with my family about my finances. | 3.54 (1.02) | 3.36 (0.99) | 3.57 (0.96) | 3.80 (0.79) | 3.87 (0.87) | 3.11 (1.23) |
| 25. | I would be motivated to have a healthier lifestyle (physical exercise, diet, vitamins, cognitive stimulation, stop smoking). | 3.91 (1.03) | 3.75 (0.99) | 3.89 (1.01) | 4.28 (0.67) | 4.08 (0.91) | 3.54 (1.34) |
| **Factor 4 Need for Knowledge** | | **4.12 (0.82)** | **4.05 (0.55)** | **4.14 (0.67)** | **4.51 (0.51)** | **4.27 (0.71)** | **3.64 (1.98)** |
| 06. | In order to decide to be tested for the presence of AD, I would need more information and details. | 3.91 (1.18) | 3.82 (1.07) | 3.69 (1.21) | 4.47 (0.65) | 4.08 (1.06) | 3.49 (1.49) |
| 07. | I would like to discuss it further and to get advice from a doctor or another health professional expert in this field. | 4.34 (0.97) | 4.39 (0.66) | 4.52 (0.74) | 4.59 (0.55) | 4.40 (0.85) | 3.77 (1.50) |
| 08. | I would like to meet a health professional, expert on genetics, in order to discuss my feelings and my thoughts. | 4.02 (1.11) | 3.71 (1.03) | 3.99 (1.04) | 4.59 (0.63) | 4.28 (0.89) | 3.55 (1.45) |
| 14. | I will be motivated to stay abreast of new developments in AD treatment and prevention. | 4.21 (0.89) | 4.28 (0.65) | 4.35 (0.78) | 4.40 (0.68) | 4.30 (0.75) | 3.75 (1.30) |

*Note*. total *N* = 650; country subsamples *n* = 130. *Range from 1 = strongly disagree to 5 = strongly agree **reverse-coded items (range from 1 = strongly agree to 5 = strongly disagree. Mean scores under 3.0 indicate stronger agreement with the items´ negatively worded statement).
