# Supplementary material for: Attitudes toward pre-symptomatic screening for Alzheimer’s dementia in five European countries: a comparison of family members of people with Alzheimer’s dementia versus non-family members
Source: Front Genet. 2023 Dec 15;14:1305107. doi: 10.3389/fgene.2023.1305107 (PMC10757380; doi:10.3389/fgene.2023.1305107)
Supplement: Supplementary file 2 [file Table2.DOCX]

Supplementary Material

Attitudes toward pre-symptomatic screening for Alzheimer’s Dementia in five European countries: a comparison of family members of people with Alzheimer’s Dementia versus non-family members

#### Ioanna Antigoni Angelidou, Marina Makri, Konrad Beyreuther, Mercè Boada, Akyllina Despoti, Sebastiaan Engelborghs, Andrea Miguel, Isabel Rodríguez, Hannah Stocker, Joke Temmerman, Magdalini Tsolaki, Görsev Yener, Deniz Yerlikaya, Birgit Teichmann^*^

*** Correspondence:** Dr. Birgit Teichmann: teichmann@nar.uni-heidelberg.de

**Supplementary Material 2**

**STROBE Statement—Checklist of items that should be included in reports of cross-sectional studies**

|  | Item No | Recommendation | Page No |
| --- | --- | --- | --- |
| **Title and abstract** | 1 | (a) Indicate the study’s design with a commonly used term in the title or the abstract | 2 |
|  |  | (b) Provide in the abstract an informative and balanced summary of what was done and what was found | 2 |
| Introduction | | | |
| Background/rationale | 2 | Explain the scientific background and rationale for the investigation being reported | 3 |
| Objectives | 3 | State specific objectives, including any prespecified hypotheses | 5 |
| Methods | | | |
| Study design | 4 | Present key elements of study design early in the paper | 5 |
| Setting | 5 | Describe the setting, locations, and relevant dates, including periods of recruitment, exposure, follow-up, and data collection | 5 |
| Participants | 6 | (a) Give the eligibility criteria, and the sources and methods of selection of participants | 5 |
| Variables | 7 | Clearly define all outcomes, exposures, predictors, potential confounders, and effect modifiers. Give diagnostic criteria, if applicable | 7,9,10 |
| Data sources/ measurement | 8* | For each variable of interest, give sources of data and details of methods of assessment (measurement). Describe comparability of assessment methods if there is more than one group | 7 |
| Bias | 9 | Describe any efforts to address potential sources of bias | 7,9,10,14,15 |
| Study size | 10 | Explain how the study size was arrived at | 5 |
| Quantitative variables | 11 | Explain how quantitative variables were handled in the analyses. If applicable, describe which groupings were chosen and why | 5,6,7 |
| Statistical methods | 12 | (a) Describe all statistical methods, including those used to control for confounding | 6,7,9,10 |
|  |  | (b) Describe any methods used to examine subgroups and interactions | 6,7,9,10 |
|  |  | (c) Explain how missing data were addressed | n.a. |
|  |  | (d) If applicable, describe analytical methods taking account of sampling strategy | n.a. |
|  |  | (e) Describe any sensitivity analyses | n.a. |
| Results | | | |
| Participants | 13* | (a) Report numbers of individuals at each stage of study—eg numbers potentially eligible, examined for eligibility, confirmed eligible, included in the study, completing follow-up, and analysed | 6 |
|  |  | (b) Give reasons for non-participation at each stage | n.a. |
|  |  | (c) Consider use of a flow diagram | n.a. |
| Descriptive data | 14* | (a) Give characteristics of study participants (eg demographic, clinical, social) and information on exposures and potential confounders | 7 |
|  |  | (b) Indicate number of participants with missing data for each variable of interest | n.a. |
| Outcome data | 15* | Report numbers of outcome events or summary measures | 7,8,9,10 |
| Main results | 16 | (a) Give unadjusted estimates and, if applicable, confounder-adjusted estimates and their precision (eg, 95% confidence interval). Make clear which confounders were adjusted for and why they were included | 9,10 |
|  |  | (b) Report category boundaries when continuous variables were categorized | 5,6 |
|  |  | (c) If relevant, consider translating estimates of relative risk into absolute risk for a meaningful time period | n.a. |
| Other analyses | 17 | Report other analyses done—eg analyses of subgroups and interactions, and sensitivity analyses | 9,10 |
| Discussion | | | |
| Key results | 18 | Summarize key results with reference to study objectives | 10 |
| Limitations | 19 | Discuss limitations of the study, taking into account sources of potential bias or imprecision. Discuss both direction and magnitude of any potential bias | 15 |
| Interpretation | 20 | Give a cautious overall interpretation of results considering objectives, limitations, multiplicity of analyses, results from similar studies, and other relevant evidence | 11,12,13,14,15 |
| Generalisability | 21 | Discuss the generalizability (external validity) of the study results | 15 |
| Other information | | | |
| Funding | 22 | Give the source of funding and the role of the funders for the present study and, if applicable, for the original study on which the present article is based | 17 |

*Give information separately for exposed and unexposed groups.

**25-item PRE-ADS (English version) ***

| **Acceptance of dementia screening and the need of knowledge** |
| --- |
| *Please, choose if you agree or disagree with the following statements* |
| 1. I would like to know if I am at higher risk than others for developing Alzheimer’s disease. |
| 2. I would like to be tested for the presence of AD on a regular basis with a short questionnaire. |
| 3. I would like to get a genetic testing with blood sample in order to find if I am in a higher risk for AD. |
| 4. I would like to be tested for the presence of AD on a regular basis with pictures of my head or brain (CT-scan or MRI). |
| 5. I would like to be tested for the presence of AD on a regular basis with the use of biomarkers in cerebrospinal fluid (Aβ amyloid, t-protein). |
| 6. In order to decide to be tested for the presence of AD, I would need more information and details. |
| 7. If I was informed that I am in a higher risk of AD, I would like to discuss it further and to get advice from a doctor or another health professional expert in this field. |
| 8. If I was informed that I am in a higher risk of AD, I would like to meet a health professional, expert on genetics, in order to discuss my feelings and my thoughts |
| **Motivations and barriers of AD biomarkers testing**** |
| *If you were informed that you are at a higher risk of developing AD, to what extent you would prefer to do the following statements (select an answer).* |
| 9. My family will suffer from the additional costs of my care |
| 10. My family will suffer emotionally |
| 11. I feel that I would be overwhelmed by mental pain |
| 12. I feel that I would be overwhelmed by intense anxiety |
| 13. I would improve my quality of life |
| 14. I will be motivated to stay abreast of new developments in AD treatment and prevention |
| 15. My family would suffer financially |
| 16. My family would suffer emotionally |
| 17. My family would have a better chance to take care of me |
| 18. I think that others will treat me in a different way |
| 19. I would be depressed |
| 20. I would be anxious |
| 21. I would give up on life |
| 22. I would have more time to plan my future |
| 23. I would have more time to talk with my family about my health care |
| 24. I would have more time to talk with my family about my finances |
| 25. I would be motivated to have a healthier lifestyle (physical exercise, diet, vitamins, cognitive stimulation, stop smoking) |

*Each item is rated on a 5-point Likert scale including “strongly agree”, “agree”, “I don’t know”, “disagree”, and “strongly disagree”. ** Each statement begins with “If I was informed that I am in a higher risk of AD…”
