# Supplementary material for: Attitudes toward pre-symptomatic screening for Alzheimer’s dementia in five European countries: a comparison of family members of people with Alzheimer’s dementia versus non-family members
Source: Front Genet. 2023 Dec 15;14:1305107. doi: 10.3389/fgene.2023.1305107 (PMC10757380; doi:10.3389/fgene.2023.1305107)
Supplement: Supplementary file 3 [file Table3.DOCX]

**PRE-ADS Questionnaire**

| **Item** | **English** | **Dutch** | **German** | **Greek** | **Spanish** | **Turkish** |
| --- | --- | --- | --- | --- | --- | --- |
| **1** | I would like to know if I am at higher risk than others for developing Alzheimer’s disease. | Ik zou graag willen weten of ik een groter risico loop dan anderen om de ziekte van Alzheimer te ontwikkelen. | Ich würde gerne wissen, ob ich ein höheres Risiko als andere habe, an Alzheimer/Demenz zu erkranken. | Θα ήθελα να μάθω αν διατρέχω υψηλότερο κίνδυνο από άλλους για εμφάνιση της ΝΑ. | Me gustaría saber si tengo mayor riesgo que otros de desarrollar la enfermedad de Alzheimer. | Başka kişilere kıyasla Alzheimer hastalığı geliştirme riskimin daha fazla olup olmadığını bilmek isterim. |
| **2** | I would like to be tested for the presence of AD on a regular basis with a short questionnaire | Ik zou graag regelmatig willen getest worden op de aanwezigheid van de ziekte van Alzheimer met een korte vragenlijst. | Ich würde gerne regelmäßig anhand eines kurzen Fragebogens auf Alzheimer/Demenz getestet werden. | Θα ήθελα να κάνω έλεγχο σε τακτικά χρονικά διαστήματα για να παρακολουθώ το αν έχω εμφανίσει συμπτώματα ΝΑ μέσα από μια σειρά ερωτηματολογίων/τεστ αξιολόγησης. | Me gustaría que me hicieran pruebas para detectar la presencia de Alzheimer de forma regular con un breve cuestionario. | Alzheimer hastalığım olup olmadığının düzenli olarak kısa bir ölçek ile değerlendirilmesini isterim. |
| **3** | I would like to get a genetic test with a blood sample to determine if I am at a higher risk for AD. | Ik zou graag regelmatig willen getest worden op de aanwezigheid van de ziekte van Alzheimer met een bloedstaal. | Ich würde gerne einen Gentest (mit einer Blutproben) machen, um festzustellen, ob ich ein höheres Risiko habe, an Alzheimer/Demenz zu erkranken. | Θα ήθελα να κάνω ένα γενετικό έλεγχο (με δείγμα αίματος) για να δω αν έχω υψηλότερο κίνδυνο εμφάνισης ΝΑ. | Me gustaría hacerme una prueba genética con una muestra de sangre para determinar si tengo un mayor riesgo de desarrollar Alzheimer. | Alzheimer hastalığı için yüksek risk altında olup olmadığımı kan örneğinden genetik test yaptırarak öğrenmek istiyorum. |
| **4** | I would like to be tested for the presence of AD on a regular basis with pictures of my head or brain (CT-scan or MRI). | Ik zou graag regelmatig willen getest worden op de aanwezigheid van de ziekte van Alzheimer met behulp van beelden van mijn hoofd of hersenen (CT-scan of MRI). | Ich würde gerne regelmäßig anhand von Bildern von meinem Kopf oder Gehirn (CT-Scan oder MRT) auf Alzheimer/Demenz getestet werden. | Θα ήθελα να κάνω έλεγχο σε τακτικά χρονικά διαστήματα για να παρακολουθώ το αν έχω εμφανίσει συμπτώματα ΝΑ με απεικόνιση του εγκεφάλου (MRI, PET-scan). | Me gustaría que me hiciera una prueba para detectar la presencia de Alzheimer de forma. regular con imágenes en mi cabeza o cerebro (tomografía computarizada o resonancia magnética). | Alzheimer hastalığım olup olmadığının düzenli olarak beyin görüntüleme (bilgisayarlı tomografi veya MR) ile değerlendirilmesini isterim. |
| **5** | I would like to be tested for the presence of AD on a regular basis with the use of biomarkers in cerebrospinal fluid (Aβ amyloid, t-protein). | Ik zou graag regelmatig willen getest worden op de aanwezigheid van de ziekte van Alzheimer door gebruik te maken van biomarkers in het hersenvocht (amyloïde-eiwit, tau-eiwitten). | Ich würde gerne regelmäßig anhand von Biomarkern im Liquor (Aβ-Amyloid, Tau-Protein) auf Alzheimer/Demenz getestet werden. | Θα ήθελα να κάνω έλεγχο σε τακτικά χρονικά διαστήματα για να παρακολουθώ το αν έχω εμφανίσει συμπτώματα ΝΑ με την χρήση βιοδεικτών στο εγκεφαλονωτιαίο υγρό (Αβ αμυλοειδές, τ-πρωτεΐνης). | Me gustaría que me hicieran pruebas para detectar la presencia de Alzheimer de forma reglar con el uso de biomarcadores en el líquido cefalorraquídeo (amiloide αβ, proteína t). | Alzheimer hastalığım olup olmadığının düzenli olarak beyin omurilik sıvısı biyobelirteçleri (Aß, tau protein) ile değerlendirilmesini isterim. |
| **6** | In order to decide to be tested for the presence of AD, I would need more information and details. | Om te beslissen getest te worden op de aanwezigheid van de ziekte van Alzheimer, zou ik meer informatie en details nodig hebben. | Um entscheiden zu können, ob ich mich auf Alzheimer/Demenz testen lassen würde, bräuchte ich mehr Informationen und Details. | Αν ήταν να κάνω έλεγχο για το αν διατρέχω υψηλότερο κίνδυνο για ΝΑ, θα ήθελα να με ενημερώσουν καλύτερα και με περισσότερες λεπτομέρειες. | Para decidir hacerme la prueba de presencia de Alzheimer, necesitaría más información y detalles. | Alzheimer hastalığı varlığı açısından test edilme kararı vermeden önce daha fazla bilgi ve detay edinmeye ihtiyacım var. |
| **7** | If I was informed that I am at a higher risk of AD, I would like to discuss it further and to get advice from a doctor or another health professional expert in this field. | Als ik geïnformeerd zou worden dat ik een verhoogd risico op de ziekte van Alzheimer heb, zou ik dit verder willen bespreken en advies willen inwinnen bij een arts of andere gezondheidsdeskundige in dit gebied. | Um entscheiden zu können, ob ich mich auf Alzheimer/Demenz testen lassen würde, bräuchte ich mehr Informationen und Details. | Αν μάθαινα ότι διατρέχω υψηλότερο κίνδυνο για ΝΑ, θα ήθελα να το συζητήσω περισσότερο και να συμβουλευτώ τον γιατρό μου ή έναν επαγγελματία υγείας ειδικό σε αυτά τα θέματα. | Si informan de que tengo un mayor riesgo de desarrollar Alzheimer, me gustaría discutirlo más a fondo y tener el consejo de un médico u otro profesional de la salud experto en el campo. | Eğer Alzheimer hastalığı açısından yüksek risk grubunda olduğum hakkında bilgilendirilirsem, bu konuyu bir doktor veya konu ile ilgili bir sağlık uzmanı ile görüşüp öneri almak isterim. |
| **8** | If I was informed that I am at a higher risk of AD, I would like to meet a health professional, an expert on genetics, in order to discuss my feelings and my thoughts. | Als ik geïnformeerd zou worden dat ik een verhoogd risico op de ziekte van Alzheimer heb, zou ik graag een gezondheidsdeskundige in het gebied van genetica ontmoeten om mijn gevoelens en gedachten te bespreken. | Wenn mir mitgeteilt werden würde, dass ich ein erhöhtes Risiko für Alzheimer/Demenz habe, würde ich dies gerne mit einer medizinischen Fachkraft, einem Experten für Genetik, unterhalten, um über meine Gefühle und Gedanken zu sprechen, | Αν μάθαινα ότι διατρέχω υψηλό κίνδυνο εμφάνισης ΝΑ, θα ήθελα να μπορώ να δω έναν εξειδικευμένο επαγγελματία υγείας για να συζητήσω τα συναισθήματα και τις σκέψεις μου. | Si me informan de que tengo un mayor riesgo de EA, me gustaría conocer a un profesional de la salud, con experiencia en genética, para discutir mis sentimientos y pensamientos. | Eğer Alzheimer hastalığı açısından yüksek risk grubunda olduğum hakkında bilgilendirilirsem, genetik alanında uzman bir sağlık çalışanı ile görüşüp duygularım ve düşüncelerim hakkında konuşmak isterim. |
| **9** | My family will suffer from the additional costs of my care. | Mijn familie zal lijden onder de extra kosten van mijn zorg. | Meine Familie würde unter den zusätzlichen Kosten für meine Pflege leiden. | Η οικογένειά μου θα επιβαρυνόταν με επιπλέον έξοδα για την περίθαλψή μου. | Mi familia sufrirá costes adicionales relacionados con mis cuidados y atención. | Ailem bakımımın getireceği ek maliyetler yüzünden sıkıntı çekecek |
| **10** | My family will suffer emotionally. | Mijn familie zal emotioneel lijden. | Meine Familie würde emotional belastet werden. | Θα επιβαρύνονταν ψυχικά η οικογένειά μου. | Mi familia sufrirá emocionalmente. | Ailem duygusal olarak sıkıntı çekecek |
| **11** | I feel that I would be overwhelmed by mental pain. | Ik voel dat ik overspoeld zou worden door mentale pijn. | Ich glaube, dass ich von seelischen Schmerzen überwältigt werden würde. | Νιώθω ότι θα κατακλυζόμουν από ψυχικό πόνο. | Siento que me sentiría abrumado por el dolor mental. | Zihinsel acıdan bunalacağımı hissediyorum |
| **12** | I feel that I would be overwhelmed by intense anxiety. | Ik voel dat ik overspoeld zou worden door intense angst. | Ich glaube, dass ich von starken Ängsten überwältigt werden würde. | Νιώθω ότι θα κατακλυζόμουν από έντονο άγχος. | Siento que me sentiría abrumado por una intensa ansiedad. | Yoğun kaygıdan bunalacağımı hissediyorum |
| **13** | I would improve my quality of life. | Ik zou de kwaliteit van mijn leven verbeteren. | Ich würde meine Lebensqualität verbessern. | Θα βελτίωνα την ποιότητα της ζωής μου. | Mejoraría mi calidad de vida. | Yaşam kalitemi arttırırdım. |
| **14** | I will be motivated to stay abreast of new developments in AD treatment and prevention. | Ik zal gemotiveerd zijn om op de hoogte te blijven van nieuwe ontwikkelingen in de behandeling en preventie van de ziekte van Alzheimer. | Ich würde motiviert sein, mich über neue Entwicklungen in der Behandlung und Prävention von Alzheimer/Demenz auf dem Laufenden zu halten. | Θα ήμουν πιο κινητοποιημένος να μάθω για νέες θεραπείες και προληπτική αντιμετώπιση της νόσου. | Estaría motivado/a para mantenerme al tanto de los nuevos desarrollos en el tratamiento y la prevención de la Alzheimer. | Alzheimer hastalığı tedavisi ve önlenmesindeki yeni gelişmelerden haberdar olmak için motive olurum. |
| **15** | My family would suffer financially. | Mijn familie zou er financieel onder lijden. | Meine Familie würde finanziell leiden. | Η οικογένειά μου θα υπέφερε οικονομικά. | Mi familia sufrirá económicamente. | Ailem maddi sıkıntı çekerdi. |
| **16** | My family would suffer emotionally. | Mijn familie zou er emotioneel onder lijden. | Meine Familie würde emotional leiden. | Η οικογένειά μου θα υπέφερε συναισθηματικά. | Mi familia sufrirá emocionalmente. | Ailem duygusal sıkıntı çekerdi. |
| **17** | My family would have a better chance to take care of me. | Mijn familie zou een betere kans hebben om voor mij te zorgen. | Meine Familie hätte eine bessere Möglichkeit, für mich zu sorgen. | Η οικογένειά μου θα είχε την δυνατότητα να οργανώσει καλύτερα την φροντίδα μου. | Mi familia tendría una mejor oportunidad de cuidarme. | Ailemin bana bakmak için daha iyi bir şansı olurdu |
| **18** | I think that others will treat me in a different way. | Ik denk dat anderen mij op een andere manier zullen behandelen. | Ich denke, dass andere mich anders behandeln werden. | Πιστεύω ότι οι άλλοι θα με αντιμετώπιζαν διαφορετικά. | Pienso que los demás me tratarían de una forma distinta. | Başkalarının bana farklı şekilde davranacağını düşünüyorum. |
| **19** | I would be depressed. | Ik zou depressief zijn. | Ich wäre deprimiert. | Θα ένιωθα μεγάλη θλίψη. | Estaría deprimido/a. | Depresif olurdum. |
| **20** | I would be anxious. | Ik zou angstig zijn. | Ich wäre ängstlich | Θα ήμουν αγχωμένος/η και θα ένιωθα ανησυχία. | Estaría ansioso/a. | Kaygılı olurdum. |
| **21** | I would give up on life. | Ik zou het leven opgeven. | Ich würde das Leben aufgeben. | Θα παραιτούμουν από την ζωή. | Renunciaría a mi vida. | Hayattan vaz geçerdim. |
| **22** | I would have more time to plan my future. | Ik zou meer tijd hebben om mijn toekomst te plannen. | Ich hätte mehr Zeit, um meine Zukunft zu planen. | Θα είχα περισσότερο χρόνο για να σχεδιάσω το μέλλον μου. | Tendría más tiempo para planificar mi futuro. | Geleceğimi planlamak için daha fazla zamanım olurdu. |
| **23** | I would have more time to talk with my family about my healthcare. | Ik zou meer tijd hebben om met mijn familie over mijn gezondheidszorg te praten. | Ich hätte mehr Zeit, um mit meiner Familie über meine Gesundheitsversorgung zu sprechen. | Θα είχα περισσότερο χρόνο να συζητήσω με την οικογένεια μου τον τρόπο περίθαλψης. | Tendría más tiempo para hablar con mi familia sobre mi atención médica. | Ailemle sağlığım hakkında konuşmak için daha fazla zamanım olurdu. |
| **24** | I would have more time to talk with my family about my finances. | Ik zou meer tijd hebben om met mijn familie over mijn financiën te praten. | Ich hätte mehr Zeit, um mit meiner Familie über meine Finanzen zu sprechen. | Θα είχα περισσότερο χρόνο να συζητήσω με την οικογένειά μου τα οικονομικά ζητήματα που μπορεί να ανακύψουν. | Tendría más tiempo para hablar con mi familia sobre el tema económico. | Ailemle mali durumum hakkında konuşmak için daha fazla zamanım olurdu |
| **25** | I would be motivated to have a healthier lifestyle (physical exercise, diet, vitamins, cognitive stimulation, stop smoking). | Ik zou gemotiveerd zijn om een gezondere levensstijl te hebben (meer lichaamsbeweging, gezonder eten, zorgen voor cognitieve stimulatie, stoppen met roken, alcohol inname matigen). | Ich wäre motiviert, einen gesünderen Lebensstil zu führen (körperliche Bewegung, Ernährung, Vitamine, geistige Stimulation, mit dem Rauchen aufhören) | Θα ήμουν κινητοποιημένος να υιοθετήσω έναν υγιεινότερο τρόπο ζωής (σωματική άσκηση, ισορροπημένη δίαιτα, βιταμίνες, νοητική άσκηση, διακοπή καπνίσματος). | Me motivaría tener un estilo más saludable (ejercicio físico, dieta, vitaminas, estimulación cognitiva, dejar de fumar...). | Daha sağlıklı bir yaşam tarzını benimsemek için daha motive olurdum (fiziksel egzersiz, diyet, vitaminler, bilişsel uyarım, sigarayı bırakmak) |
